# High-ZT Due to the Influence of Copper in Ti(Ni_1-*x*_Cu*_x_*)Sn

**DOI:** 10.3390/ma16051902

**Published:** 2023-02-25

**Authors:** Yatir Sadia, Dan Lumbroso, Yaniv Gelbstein

**Affiliations:** 1 The Department of Materials Engineering, Ben-Gurion University of the Negev, Beer-Sheva 8400711, Israel; 2 Nuclear Research Center of the Negev, NRCN, Beer-Sheva 8400711, Israel

**Keywords:** half-Heusler, TiNiSn, thermoelectric, arc melting

## Abstract

Most high-performance thermoelectric materials require either expensive, rare, or toxic elements. By doping TiNiSn, a low-cost, abundant thermoelectric compound, with copper as an n-type donor, some optimization can be performed for such materials. Ti(Ni_1-*x*_Cu*_x_*)Sn was synthesized by arc melting followed by heat treatment and hot pressing. The resulting material was analyzed for its phases using XRD and SEM and its transport properties. Cu undoped and 0.05/0.1% doped samples showed no additional phases in addition to the matrix half-Heusler phase, while the 1% copper doping initiated some Ti_6_Sn_5_ and Ti_5_Sn_3_ precipitation. The transport properties showed that copper acts as an n-type donor while also lowing the lattice thermal conductivity of the materials. the sample containing 0.1% copper showed the best figure of merit, ZT, with a maximal value of 0.75 and an average value of 0.5 through 325–750 K showing a 125% improvement over the undoped sample of TiNiSn.

## 1. Introduction

In the past few decades, the need for energy-saving devices based on low-cost and abundant materials has been more and more evident. Several technologies for the production of such energy, such as solar, wind, and geothermal, have received a large boost in research and development. In addition, devices that can convert some of the wasted energy from heat back into useful energy have also attracted attention. One such type of device is a thermoelectric generator. Thermoelectric generators convert heat from a wasted resource into useful electricity. This is performed by straining n-type and p-type thermoelectric materials such that they are in series electrically but in parallel thermally.

Thermoelectric materials take advantage of the Seebeck effect in degenerate semiconductors [1] with charge carriers, electrons in n-type, and holes in p-type semiconductors, drifting from the hot side of the semiconductor to the cold side of the semiconductor. Maximizing the potential of thermoelectric materials requires tuning three major transport properties, the Seebeck effect to increase the amount of charge movement, the electrical conductivity to reduce joule losses, and the thermal conductivity to reduce the amount of thermal energy that goes unused. The simplest indicator for thermoelectric performance is the dimensionless figure of merit, ZT, with T being the temperature in K and Z = α^2^/ρκ, where α is the Seebeck coefficient, ρ is the electric resistivity, and κ is the thermal conductivity. Another performance indicator is the power factor, which is calculated by PF = α^2^/ρ; the power factor is much more important in the application where maximum power is required rather than maximum efficiency, usually when the heat source is large.

As mentioned before, the best thermoelectric materials are materials that exhibit a high Seebeck coefficient and low electric resistivity with low thermal conductivity, which are degenerate semiconductors with low thermal conductivity. The main way to find such materials is to use semiconductors with either heavy elements or complex unitcells and microstructures. Several of the best such materials are chalcogenides such as PbTe and Bi_2_Te_3_ [2,3], silicides such as Mg_2_Si and Mn_4_Si_7_ [4,5], skutterudites such as Co_4_Sb_12_ [6,7], and half-Heusler (HH) materials such as TiNiSn [8,9]. One of the biggest advantages of half-Heusler is that, unlike many thermoelectric materials, they do not contain any toxic elements such as lead and any rare elements such as tellurium or germanium. Half-Heuslers are based mainly on TiNiSn, all of which are common materials. Half-Heusler materials are intermetallic compounds that have unique properties. The general formula is XYZ, where X and Y are transition metals and Z is a p-block metal of semimetal. They consist of a cubic structure of four intertwined FCC structures where X, Y, and Z each make up one FCC, and the fourth structure is made up of vacancies [10].

**Figure 1:**
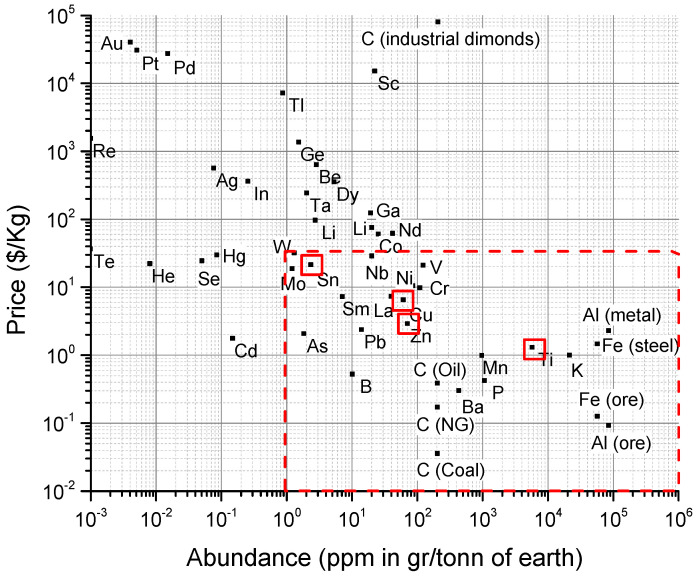
Abundance and price of elements with Ti, Ni, Sn, and Cu marked data from Hurd et al. [11].

The most common alloying elements on the X site in half-Heusler materials (i.e., Ti) are zirconium and hafnium, which are expensive and rare, whereas the base TiNiSn is much less expensive. Therefore, finding a low-cost dopant, such as in the form of copper on the nickel site, could allow for lower-cost thermoelectric materials. In Figure 1, the price and abundance of some elements are shown, highlighting the very low price and high abundance of titanium, tin, nickel, and copper [11]. The rule for HH compounds is that the first element gives its outer electrons to allow the second element to fill its d orbital, with the third element filling its outer shell. This is called the 18-electron rule [12] and is configured in Ti^4+^Ni^0^Sn^4-^ oxidation states. Copper already has its d orbital full with a single excess electron. As such, it is expected to contribute its excess electron to the system as an n-type dopant.

Several attempts were used to add 2.5–25% copper [13,14,15,16], with various results reaching a ZT of about 0.6 for pure TiNiCu*_x_*Sn and about 0.8 for (Ti, Zr, Hf) NiCu_x_Sn [16]. It is worth noting that in all those attempts, the addition was as an additional element with the TiNiCu*_x_*Sn formula, with copper being assumed to sit at the vacancy site. However, some publications also show that some of the copper sits at grain boundaries [15]. It is noteworthy that over 15% additional copper does start showing a typical full-Heusler peak in X-ray diffraction, XRD [13,15]. It is unclear whether this is due to copper sitting in the vacancy site or copper sitting in the nickel site and allowing for some excess nickel to form the full-Heusler. One study on copper as a dopant on the nickel site [17] has not reported the electronic properties above 400 K, the thermal conductivity, as well as the microstructure or XRD analysis. However, copper was shown to turn TiNiSn into a more conductive *n*-type material.

In this study, the effect of copper in the form of TiNi_1-*x*_Cu_*x*_Sn on the thermoelectric properties of half-Heusler thermoelectric materials is tested to try and optimize the electronic properties of these materials. By doping to small amounts of copper, some tuning of the charge carrier concentration can allow us to optimize the electronic properties of this system. This will allow for later using microstructure control to reduce the thermal conductivity of the system through the phonon glass approach in later studies.

## 2. Materials and Methods

Raw metals of Ti (5N), Ni (5N), Sn (5N), and Cu (5N) were weighed to produce 20 g of TiNi_1-*x*_Cu*_x_*Sn with *x* = 0.0005, 0.001 and 0.01 of each composition by melting 5-g buttons in each composition using a homebuilt arc melting device. Each button was melted 4 times under argon atmosphere to provide maximum homogeneity. High-purity argon was achieved by melting a titanium getter beforehand. The resulting buttons were milled to 125 µm powder in an agate mortar and pestle and pressed into a pellet at 300 MPa. The pellets were covered by a tantalum foil and sealed at high vacuum (5 × 10^−5^ torr) in a quartz ampule. The samples were then heat treated at 1163 K for 100 h.

The samples’ crystal structure was analyzed by X-ray powder diffraction. X-ray diffraction (XRD) data were collected using a Panalytical Empyrean powder diffractometer (Melvern Panalytical, Melvern, UK) equipped with a position-sensitive X’Celerator detector using Cu Kα radiation (λ = 1.5405 Å) operated at 40 kV and 30 mA. Diffraction pattern was taken before the heat treatment directly after arc melting as well as after the heat treatment and after hot pressing the samples.

The samples were hot-pressed in a commercial hot press (FCT systeme GmbH, Frankenblick, Germany) using a 30 mm graphite die. The die was sprayed with boron nitride spray to prevent sticking of the sample, and graphite foils were placed above and below the sample to release stress. The pressing sequence included pressureless heating to 473 K, 60 min dwell time at 473 K, heating at 10 K/min to 1273 K with 50 MPa pressure, 60 min dwell time at 1273 K under 50 MPa, and cooling 10 K/minute with pressure being brought down to 3 MPa at room temperature. XRD samples were taken before and after hot-pressing. Due to the low melting point of tin (503 K), any leftover tin will cause the sample to melt during the hot press process and spill out of the die. As such, the extra thermal treatment at 473 K for 60 min is added to react with any leftover tin 30 K below the melting point before using any pressure.

Density measurements of all samples showed 97% of the theoretic density or above using the Archimedes method, where the theoretic value was taken from theoretical XRD data as 7.21 g/cm^3^, with the changes in density due to copper additions being negligible.

The samples were cut with a diamond saw for thermal conductivity, Seebeck coefficient, and electrical resistivity measurements, and additional testing such as scanning electron microscopy—SEM. The microstructure was analyzed by scanning electron microscopy (SEM), while the chemical analysis was performed using energy dispersive X-ray spectroscopy (EDS) (JEOL-7400F, Tokyo, Japan).

Electronic transport properties*:* Seebeck coefficient and the electrical resistivity were measured by Linseis LSR-3/800 Seebeck coefficient/electrical resistance measuring system (Linseis Messgeräte GmbH, Selb, Germany) up to ~723 K with a Δ5 K difference to calculate the Seebeck coefficient.

Thermal transport properties: The thermal conductivity was measured from room temperature up to 723 K using the flash diffusivity method on 10 × 10 samples covered in graphite spray using an LFA 457 microflash, (Netzsch GmbH, Selb, Germany). Thermal conductivity (κ) values were calculated using the equation κ = β·δ·C_p_ where β is the thermal diffusivity, C_p_ is the specific heat measured using differential scanning calorimetry, STA 449-(Netzsch GmbH, Selb, Germany), and δ is the bulk density of the sample calculated using the Archimedes method.

## 3. Results

### 3.1. Phase Identification

**Figure 2:**
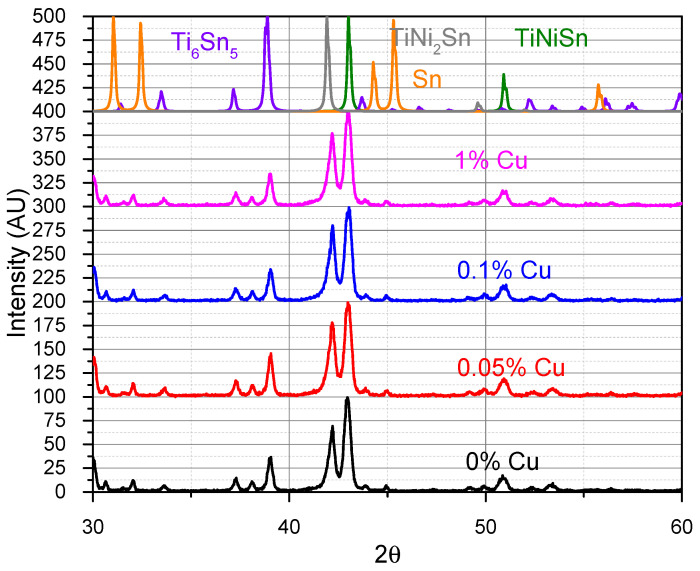
XRD of the TiNi_1-*x*_Cu*_x_*Sn directly after arc melting, showing mainly TiNiSn, TiNi_2_Sn, and Ti_6_Sn_5_. Black—0% Cu, Red—0.05% Cu, Blue—0.1% Cu, Pink—1% Cu. Theoretical phases—Green TiNiSn, Gray—TiNi_2_Sn, Orange—Sn, Purple Ti_6_Sn_5_.

Figure 2 shows the XRD directly after arc melting. The resulting phases were TiNiSn, TiNi_2_Sn, and Ti_6_Sn_5_ with a negligible free Sn. This is expected from the Ti-Ni-Sn phase diagram [18], with TiNi_2_Sn having a much higher melting point than TiNiSn and, therefore, precipitating out of the melt at higher temperatures near 1720 K. Ti_6_Sn_5_ similarly precipitates at 1763 K. During cooling TiNiSn is formed near 1455 K with a reaction between the TiNi_2_Sn with Ti_6_Sn_5_ and small amounts of leftover Sn. However, due to the rapid cooling in the arc-melting, this reaction does not complete and requires additional heat treatment.

**Figure 3:**
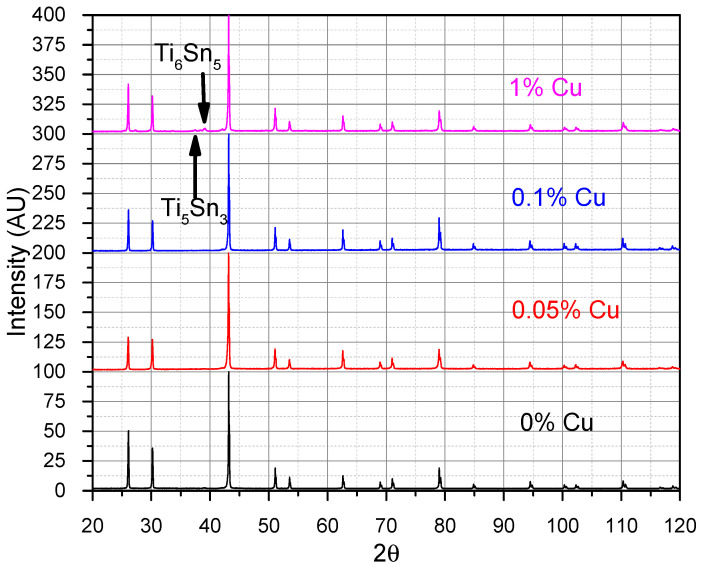
XRD of the TiNi_1-*x*_Cu*_x_*Sn after heat treatments showing a single phase up to 1% Cu, with the Ti_6_Sn_5_ and Ti_5_Sn_3_ phases marked. Black—0% Cu, Red—0.05% Cu, Blue—0.1% Cu, Pink—1% Cu.

**Figure 4:**
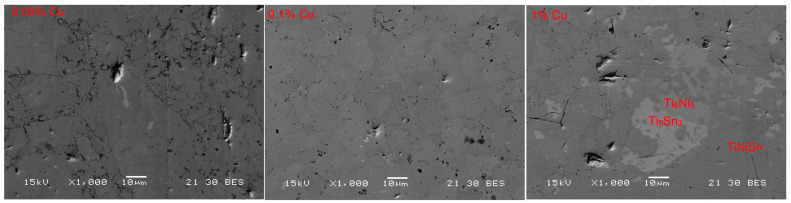
Backscattered SEM image of the 0.05%, 0.1%, and 1% Cu-doped samples.

After heat treatment at 1163 K for 100 h, only the TiNiSn HH phase was seen in the samples. In Figure 3, the samples of 0–1% Cu can be seen showing a single phase of TiNiSn with a small amount of the Ti_6_Sn_5_ and Ti_5_Sn_3_ phases in the 1% sample. This might be an indication that the amount of copper that can sit on the Ni site (i.e., the solubility limit) is very low. If the Cu is indeed sitting at a vacancy site instead of the Ni site there will be 1% nickel missing initiating the nucleation of some Ti_6_Sn_5_ and Ti_5_Sn_3_. These phases have been inferred by EDS results. The backscattered SEM image shows some Ti_6_Sn_5_ as well as Ti_5_Sn_3_ precipitates, which were measured by EDS and marked in Figure 4 for the 1% sample. No similar phases were found for the 0.05%- and 0.1%- Cu-doped samples. This might also indicate that there is a 0.2% Sn; however, this has not been seen by SEM or XRD and is undetectable in these methods.

### 3.2. Transport Properties Measurements

**Figure 5:**
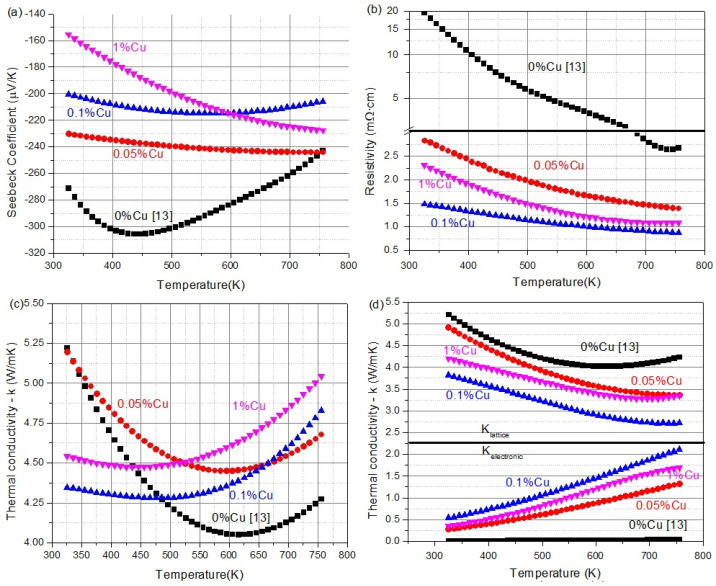
The transport properties of the currently investigated 3 compositions with 0.05%, 0.1%, and 1% Cu doping with the 0% Cu sample taken from K. Chen et al. [13] for reference. (**a**) the Seebeck coefficient, (**b**) resistivity, (**c**) thermal conductivity, and (**d**) separating into electronic and lattice thermal conductivities.

In Figure 5a–c, the transport properties, Seebeck coefficient, electrical resistivity, and thermal conductivity of the samples are shown. In Figure 5d, the thermal conductivity is split into the photonic (lattice) contribution versus the electronic contribution to the thermal conductivity. The electronic thermal conductivity was calculated using the Wiedemann–Franz law seen in Equation (1), where *κ_electronic_* is the electronic part of the thermal conductivity, σ is the electrical conductivity, *L* is the Lorentz number given by Equations (2), and *T* is the temperature in kelvin. *L* is given in Equation (2), with *k_B_* being the Boltzmann constant and *e* being the elementary charge. Finally, the lattice part was calculated by subtracting the electronic thermal conductivity from the total conductivity.

(1) $$\kappa_{electronic}=LT\sigma$$

(2) $$L=\frac{\pi^{2}}{3}{\left(\frac{k_{B}}{e}\right)}^{2}=2.44\times 10^{-8}V^{2}K^{-2}$$

From Figure 5a, the negative Seebeck coefficient values indicate the n-type nature of all the investigated compositions. A clear trend of absolute Seebeck coefficient values decreasing, with increasing the Cu doping amount, at low temperatures, can be seen as is expected upon increasing the charge carriers upon the electron donor Cu doping. The behavior of the samples is changing, with the 1% sample overtaking the 0.1% sample after 600 K. This could be due to the secondary phases in the 1% sample. The electric resistivity of the samples, seen in Figure 5b, follows a similar trend with lower resistivity with more Cu additions; however, 1% Cu is again outside this trend with higher resistivity than the 0.1% sample. Finally, the thermal conductivity, in Figure 5c, shows that with low charge carriers (0 and 0.05%), a similar trend emerges, whereas, with higher charge carriers, the inflection point of the thermal conductivity is lowered by about 100 K showing a more metallic behavior as expected. In Figure 5d, it can be seen that copper addition lowers the lattice’s thermal conductivity. This is not surprising as both alloying and doping are expected to break some symmetry; however, it is a greater change than expected with such a low doping rate. This might be attributed to an additional disordering due to the fact that a copper atom is not necessarily occupying the nickel site as nickel and copper are very similar in size and should offer very similar thermal conductivity through them.

From Figure 5, it can be seen that the temperature in which the intrinsic conduction starts to dominate goes up to higher temperatures with increasing copper content, with this temperature being about 550 K for that sample doped with no copper, as seen by the minimum in both the Seebeck in Figure 5a and the thermal conductivity in Figure 5c. This is an indication that the amount of charge carriers needed to overcome the base amount is very small, which fits the fact that this sample is not very conducive while acting like a semiconductor. However, samples with higher copper content have their Seebeck coefficient reaching a maximum at higher temperatures indicating a higher amount of charge carriers which agrees with the lower resistivity. It is worth noting that all samples behave like semiconductors, with their resistivity decreasing over the temperature range. This means that further increasing the carrier concentration, maybe with a dopant with a higher solubility than copper, can further lead to reaching the degenerate state with a very high carrier concentration and increasing the resistivity with a rise in temperature. In Figure 5d, it can be seen that the electronic part keeps increasing as expected for a semiconductor, and the phonon part of the conductivity keeps decreasing. It is also worth noting that the phonon part is very high, which means that further improvement might be achieved by introducing additional phonon scattering centers such as grain boundaries, twinning, secondary phases, and more.

**Figure 6:**
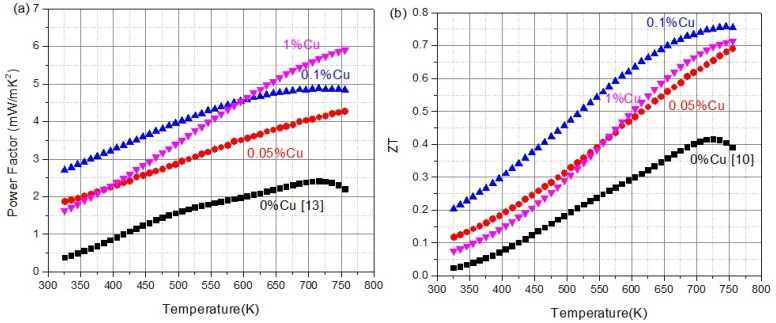
(**a**) Power factor of the (**b**) ZT of TiNi_1-*x*_Cu*_x_*Sn with a maximal ZT of 0.75 and average ZT of 0.5 from 325 to 750 K for the 0.1% Cu-doped sample [13].

Finally, the power factor and ZT of the samples are shown in Figure 6a,b, respectively. The power factor tops up at 6 (mW/mK^2^) for the 1% sample and is about two and a half times (145% improvement) the maximal temperature. However, the average power factor is for the 0.1% sample over this temperature range, with 2.5 times the average power factor (151% improvement). The ZT reaches high values of up to 0.75, with the 0.1% Cu doped sample being the highest performing sample topping the undoped sample at 81% improvement at the max. The most surprising is that the ZT of this sample is higher at all the investigated temperature ranges, showing an average ZT of 0.5 over this entire range, indicating over 125% improvement over the undoped sample.

## 4. Discussion

**Figure 7:**
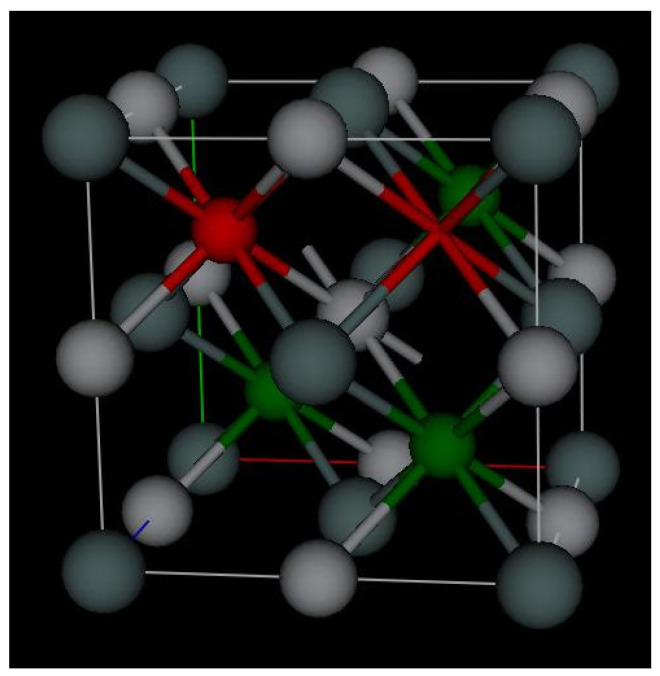
The crystal structure of TiNiSn with one of the nickel sites marked in red as a potential copper site and another vacancy site marked in red as another potential copper site image produced using Gsas 2.0 [20].

In Figure 7, we can see marked in red both one of the half-Heusler nickel sites and one of the vacant sites of the Heusler phase, which are the expected locations for copper atoms. Copper is much more soluble when it is added in the form of TiNiCu_*x*_Sn vs. TiNi_1-*x*_Cu_*x*_Sn with x = 1% already exceeds the solubility limit of TiNiSn, creating nickel-deficient secondary phases [13,16]. This is an indication that little to no copper is sitting at the normally occupied nickel site.

However, other sites for the copper atom must also be considered. As explained earlier, several papers [13,16] discuss the possibility of empty nickel sites to host copper atoms. From transmission electron microscopy, copper is shown to be sitting at the grain boundary [15] in much higher amounts than inside the grain. However, a uniform distribution was found using atom probe tomography [14]. Further analysis is important to locate which is the more stable site, the pre-occupied nickel site, the empty nickel site, the grain boundaries, or some combination of the three.

It is clear either way that copper is an electron donor and can provide a simple and cheap way to tune the electronic properties of TiNiSn. In addition, up to 0.1% copper decreases the lattice thermal conductivity, which is another indicator that the copper atom location can change the phonon dispersion and that future studies as to the copper’s incorporation are interesting. The high effect of the copper on the lattice thermal conductivity might allow for even further improvement of this system with a better understanding of this effect.

This effect was not seen by just reducing the nickel content, as was previously reported [19], where it was also stated that reducing the Ni content lowers both the thermoelectric power factor and ZT, with the main difference being the much lower resistivity and lower thermal conductivity of the copper doped samples. It is also worth noting that the biggest contributor to the lower ZT is the lattice thermal conductivity, which can be remedied in other ways, such as smaller grain sizes, twining, nanostructures, and secondary phases. These approaches could be used without a major hindrance to the electronic properties using the phonon glass electron crystal approach. Both further investigation and further optimization of the TiNiSn doped with copper are worth investigating based on these results showing a large promise for the future.

## 5. Conclusions

Small additions of copper can greatly improve the thermoelectric properties of TiNiSn. Copper in small amounts (0.1%) does not promote the formation of a secondary phase and can allow for optimization of the thermoelectric properties by adding n-type donors. Higher additions of copper (1%) promote a secondary phase of Ti_6_Sn_5_ and Ti_5_Sn_3_. Copper-doped TiNiSn marks a thermoelectric material that can reach a maximal ZT of 0.75 and an average ZT of 0.5 without using any rare, toxic, or expensive elements.

## Data Availability

Not applicable.
